# Postoperative wound complications and systemic recurrence in breast cancer

**DOI:** 10.1038/sj.bjc.6604004

**Published:** 2007-10-30

**Authors:** B L Murthy, C S Thomson, D Dodwell, H Shenoy, J S Mikeljevic, D Forman, K Horgan

**Affiliations:** 1 Department of Surgery, The General Infirmary at Leeds, Leeds LS1 3EX, UK; 2 West Midlands Cancer Intelligence Unit, Public Health Building, University of Birmingham, Birmingham B15 2TT, UK; 3 Department of Clinical Oncology, The General Infirmary at Leeds, Leeds LS1 3EX, UK; 4 Department of Acute Medicine, St James's University Hospital, Beckett's Street, Leeds LS9 7TF, UK; 5 Centre for Epidemiology and Biostatistics, University of Leeds, and the Northern and Yorkshire Cancer Registry and Information Service, Leeds LS16 6QB, UK

**Keywords:** breast cancer, wound healing, prognosis, breast surgery

## Abstract

Many factors involved in wound healing can stimulate tumour growth in the experimental setting. This study examined the relationship between wound complications and the development of systemic recurrence after treatment of primary breast cancer. One thousand and sixty-five patients diagnosed with operable primary invasive breast cancer between 1994 and 2001 were assessed for development of systemic recurrence according to whether or not a wound complication occurred after surgery, with a median follow-up of 54 months (range 15–119). There were 93 wound complications (9%). There was a statistically significant greater risk of developing systemic recurrence in patients with wound problems than those without (hazard ratio (HR) 2.87; 95% CI: 1.97, 4.18; *P*<0.0001). This remained in a multivariate analysis after adjustment for case mix variables, including Nottingham Prognostic Index (NPI) and oestrogen–progesterone receptor status (HR: 2.52; 95% CI: 1.69, 3.77; *P*<0.0001). In the good prognostic NPI group, 4 out of 27 patients (15%) with wound problems *vs* 11 out of 334 (3%) without wound problems developed systemic recurrence. The corresponding figures were 10 out of 35 (29%) *vs* 48 out of 412 (12 %) in the moderate prognostic group and 18 out of 29 (62%) *vs* 75 out of 199 (38%) in the poor prognostic group. In 29 patients NPI could not be calculated. Smokers at the time of diagnosis were more likely to develop metastatic disease than the non-smokers (HR: 1.50; 95% CI: 1.04, 2.15; *P*=0.03) after adjustment for other factors. The results suggest that patients with wound complications at primary surgery have increased rates of systemic recurrence of breast cancer.

Experimental work suggests that many factors involved in wound healing can stimulate tumour growth. Factors studied in this setting include TNF*α*, VEGF, epidermal growth factor, fibroblast growth factor, PDGF and pro-inflammatory cytokines such as interleukins 1 and 6 (Dvorak *et al*, 1995; Abramovich *et al*, 1999; Balkwill and Mantovani, 2001; Coussens and Werb, 2002). Dvorak (1986) described cancers as ‘wounds that do not heal’. As far as we are aware, there is no published evidence on the influence of delayed wound healing on the systemic recurrence rate in breast cancer.

The incidence of wound complications after breast cancer surgery ranges from 6 to 30% (Sorenson *et al*, 2002; Tran *et al*, 2003; Hall and Hall, 2004) and is increased if there is associated axillary surgery. The aim of this study was to examine the relationship between wound complications and systemic recurrence after excision of primary operable breast cancer.

## PATIENTS AND METHODS

The records of all patients with primary invasive breast cancer diagnosed under the care of a single surgeon (KH) in a specialist breast care unit from February 1994 to December 2001 were retrospectively identified from cancer registry, pathology and radiology databases. Information was extracted from case notes of the patients by a single abstractor (BLM). An independent audit of a random sample of 20 re-extracted notes was undertaken (HS) and the quality of original data extraction examined. The audit showed three minor disagreements on lymphovascular invasion and total tumour size, including background *in situ* disease but was otherwise in agreement with the main database. Patients with bilateral tumours, with initial systemic metastases, and having primary endocrine treatment for more than 1 year before palliative surgical treatment were excluded from the study. However, patients who had neo-adjuvant chemotherapy followed by surgical treatment, were included. For the purpose of this study, a wound complication was defined as any wound breakdown that occurred before the completion of adjuvant chemotherapy and radiotherapy and that needed surgical debridement, dressing, or packing or any persistent discharge from the wound. Erythema alone of the wound was not included.

The study comprises patients with primary operable breast cancer admitted under the care of one NHS consultant and managed by a team including consultants of different disciplines, such as oncology, and also trainees and so represented a typical NHS teaching hospital scenario. All surgeries were performed by the consultant or by a trainee under supervision.

Patients were routinely followed up three-monthly for a year, six-monthly for 5 years and then annually. Patients not seen in the clinic during 2003 were followed up through their General Practitioner, and their notes in the Oncology Centre were checked for evidence of any recurrence. Systemic recurrence was defined as any recurrence away from the breast, axillary or internal mammary node regions. Data on deaths were extracted from the Northern and Yorkshire Cancer Registry and Information Service in June 2004. The censoring date for data collection was 31 January 2004. This was taken as the date the patients were last known to be alive and without symptomatic disease, unless they were found to be genuinely lost to follow-up. As this was a retrospective case note audit, formal ethical approval was not required. The funding source for this study was the Breast Cancer Research Action Group and the Leeds Teaching Hospital NHS trust provided salary support for the authors (KH, DD, BLM and HS); neither of them had a role in study design, data collection, analyses, interpretation, and writing of the report or on the decision to submit for publication.

## STATISTICS

Associations between factors of interest and whether or not a patient had a wound complication were examined using the *χ*^2^-test of association.

Systemic recurrence-free survival analysis was performed with the end point being the development of systemic disease. Patients were censored at death for those who had not recurred systemically, at the date last seen in the clinic, if this was during 2003, or at 31 January 2004 for those patients last seen in the clinic before 2003, unless the patients had been lost to follow-up. The starting point was taken as the date of first breast surgery. Kaplan–Meier survival analysis was performed to plot survival curves and to estimate point estimates of 5-year survival from the date of the first operation. Equality of the survival curves was assessed using the Log Rank test. The difference in 5-year systemic recurrence-free survival between the patients having wound complications and those who did not was calculated along with an estimate of its standard error from the Kaplan–Meier analyses. The s.e. for the difference was calculated by taking the square root of the sum of the squared s.e.'s obtained for each of the 5-year survival estimates for the two groups of patients under the assumption of independence of the two groups. The numbers at risk in both the ‘wound complication’ and ‘no wound complication’ groups are also presented.

Both univariate and multivariate hazard ratios of systemic recurrence were obtained using Cox's proportional hazards models. The patient factors examined were age group at first surgery, co-morbidity (diabetes, angina, hypertension, peripheral vascular disease, renal, chronic skin problems), diabetes alone and whether the patient was a smoker at the time of diagnosis. The tumour factors were the Nottingham Prognostic Index (NPI) (Elston and Ellis, 1991) calculated from histological grade 1, 2 or 3+Nodal status (no positive nodes=1, 1–3 nodes=2 and >3 nodes positive=3)+0.2 × size of the tumour in cm, oestrogen–progesterone receptor (ER/PR) positivity status and node positivity status. Finally, the treatment factors were type of initial definitive breast surgery (wide local excision (WLE) or mastectomy), final primary breast cancer surgery, breast conservation or mastectomy, use of radiotherapy, use of chemotherapy and use of hormone therapy. Axillary node surgery does not include sentinel node procedures during the time frame of the series. Additionally, the occurrence of a wound complication following surgery was examined.

The univariate hazard ratios were obtained by including each factor separately in a different Cox's proportional hazards model. A multivariate Cox model containing only the main effects for these factors was then obtained by forcing all of the factors into the model. All analyses were performed using SPSS version 10.1.4. All the analyses were considered statistically significant when the *P*-value was less than 0.05.

## RESULTS

### General characteristics

A total of 1105 patients diagnosed with operable breast cancer between February 1994 and December 2001 under the care of a single surgeon were identified. Patients with bilateral tumours (33), with initial systemic metastasis (1), those having only axillary surgery (1) and those having primary endocrine treatment for more than 1 year before palliative surgical treatment (5) were excluded from the study. Thus, 1065 patients with operable primary breast cancer were included and they received 1356 operations in total. Initially, 426 women underwent mastectomies, 478 women WLE and 161 women had open diagnostic biopsies. Of these open biopsies, 81 women had a subsequent mastectomy, 76 had a subsequent WLE and 4 women no further surgery. Thus, the initial definitive surgery was breast conservation in 558 (52%) and mastectomy in 507 (48%) women. However, a total of 86 women having WLE needed a completion mastectomy (10 of whom had originally had an open biopsy), and so the final status of the breast was conserved in 472 (44%) and 593 (56%) patients who had a mastectomy. The minimum follow-up duration from the date of first surgery was 15 months with a median follow-up of 54 months (range 15–119 months). There were 13 (1%) patients who were lost to follow-up (3 went abroad, 3 went out of region and 7 could not be traced), with a median follow-up of 8 months (range 0–50).

The age at first surgery ranged from 22 to 98 years (median age 58 years). Of the 1065 patients, 228 (21%) had a poor NPI score, 447 (42%) had a moderate score and 361 (34%) were in the good NPI group. There were 29 (3%) patients for whom the NPI could not be calculated (10 patients underwent a mastectomy and 15 had breast conservation but did not have any axillary surgery, 2 had axillary surgery but the numbers of nodes removed/examined was not known and the other 2 had neo-adjuvant treatment before surgery for the residual tumour). The size of the tumour ranged from 1 to 220 mm, with a median of 20 mm. There were 857 patients (81%) who were ER or PR positive, and 487 patients (46%) had metastatic involvement of their axillary lymph nodes.

Ninety-three (9%) patients had wound complications (7% of the total 1356 operations). Of these, 70 involved mastectomy wounds (53 after initial mastectomy and 17 after initial breast surgery and subsequent mastectomy), 12 involved WLE wounds and 11 the separate axillary dissection wound associated with WLE to the breast. There was a total of 73 breast reconstructions; 4 of these patients had wound complications. The number of contralateral breast cancers was 4 in the 93 patients having wound complications and 29 in the 972 patients without wound complications. There was no significant delay in the administration of systemic adjuvant treatment for the patients who developed wound complications if they required such treatment.

Table 1 shows a significantly increased incidence of wound problems in smokers than in non-smokers (15 *vs* 7%; *P*=0.001); for diabetics compared to those without diabetes (20 *vs* 8%; *P*=0.03) and for those patients having a mastectomy compared with breast conservation (12 *vs* 5%; *P*<0.001) as the final breast surgery.

### Follow-up

There were 172 patients with a systemic recurrence diagnosed by 31 January 2004, and of these 115 patients had died of metastatic disease. There were also 45 non-cancer-related deaths in patients who had not developed metastatic disease. There were 21 patients who had isolated local recurrence (2%), and of these 4 patients later developed systemic metastases. The 5-year systemic recurrence-free survival estimate for all patients was 82.2% (95% CI: 79.6, 84.9), with corresponding figures for the NPI groups of 95.6% (93.1, 98.2) for the good prognostic group, 85.9% (82.2, 89.6) in the moderate prognostic group and 53.8% (46.3, 61.3) in the poor prognostic group.

**Figure 1:**
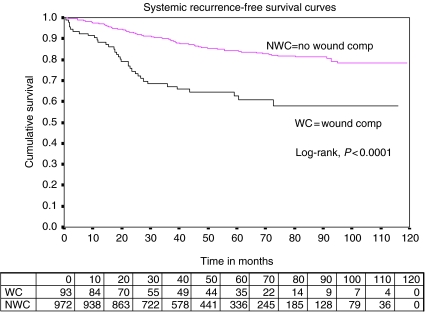
Systemic recurrence-free survival curves (Kaplan–Meier by occurrence of wound complication).

When univariate Cox regression models were fitted (Table 2), the factors that significantly affected the chances of developing a systemic recurrence in the univariate analyses were NPI score, ER/PR status, type of final breast surgery, use of radiotherapy, use of chemotherapy and use of hormone treatment. Age at first surgery, any co-morbidity and smoking were not significant in the univariate analyses. However, there was also a statistically significant relationship between the occurrence of wound complication after breast cancer surgery and the development of later systemic recurrence (*P*<0.0001). There was a difference of 21.5% (10.7, 32.3) in 5-year systemic recurrence-free survival figures for those patients not having a wound complication (84.2%; 95% CI: 81.5, 86.8) compared with 62.7% (52.2, 73.1) for those who did (Figure 1). The corresponding hazard ratio for developing a systemic recurrence was 2.87 (95% CI: 1.97, 4.18) for those having a wound complication (34 out of 93; 37%) relative to those who did not have a wound complication (138 out of 972; 14%).

**Figure 2:**
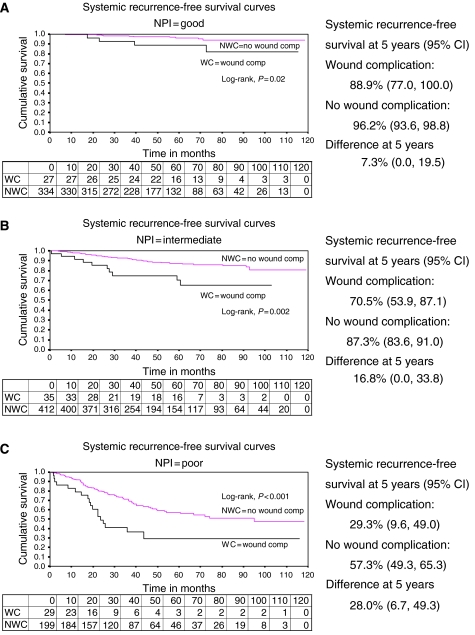
(**A**) Systemic recurrence-free survival curves (Kaplan–Meier by occurrence of wound complication) for good NPI. (**B**) Systemic recurrence-free survival curves (Kaplan–Meier by occurrence of wound complication) for intermediate NPI. (**C**) Systemic recurrence-free survival curves (Kaplan–Meier by occurrence of wound complication) for poor NPI.

This relationship remained after adjustment for case mix variables, including NPI status and ER/PR status in the multivariate model (hazard ratio 2.52; 95% CI: 1.69, 3.77; *P*<0.0001; Table 2). Figure 2A–C shows that this relationship is generally maintained across all the NPI groups, with those patients not having postoperative wound complications having better systemic recurrence-free survival times than those who did have wound complications, for each of the NPI groups. The corresponding 5-year systemic recurrence-free survival estimates are also shown in Figure 2. Despite there appearing to be differences in the proportions of patients having a systemic recurrence with and without postoperative wound complications for the different NPI groups (Table 3), the interaction between the NPI status factor and the wound complication factor was not statistically significant in the multivariate model (*P*=0.78) when the 29 cases with NPI status unknown were excluded from the analyses, showing that the factors acted independently on the hazards of developing a systemic recurrence.

Multivariate modelling revealed that NPI status and ER/PR status both independently and significantly affected the chances of developing metastatic disease in addition to the deleterious effect seen for patients having a wound complication (Table 2). There was also a borderline significant effect for type of final breast surgery (*P*=0.03), with patients who had a mastectomy being at higher risk of developing a systemic recurrence than those who had breast conservation. Similarly, those patients who smoked at diagnosis also had a slightly higher risk of developing metastatic disease (*P*=0.03).

## DISCUSSION

Wound complications after excisional surgery for primary breast cancer are not uncommon, particularly when surgery includes axillary node dissection. This study demonstrates a statistically significant relationship between the occurrence of a wound complication and the subsequent rate of development of systemic breast cancer recurrence. The magnitude of this deleterious effect (three-fold increase risk in the risk of developing metastatic disease) is of importance because it is similar to the size of benefit seen by giving adjuvant treatment to women with early breast cancer; for example, the effect of giving polychemotherapy after surgery to those with ER poor tumours is 0.61 compared with those not given it (Early Breast Cancer Trialist Collaborative Group, 2005a) or giving radiotherapy after breast-conserving surgery is 0.31 compared with those not given it (Early Breast Cancer Trialist Collaborative Group, 2005b).

It is of interest that locoregional recurrence was not increased by the occurrence of a wound complication and remained low for the total patient cohort. The putative deleterious influence of a wound complication appears to relate to systemic factors. The fact that smokers were more likely to develop wound complications, but also more likely to develop a systemic recurrence, does not explain the observed effect of those patients having a wound complication being more likely to recur systemically, given that both factors were significant in the multivariate model. This suggests that they both increase the likelihood of getting metastatic disease in the presence of the other factors. The interaction between the two factors was not significant (*P*=0.72), indicating that the effect of developing a systemic recurrence for those patients having a wound complication compared to those who did not was the same whether or not the patient smoked at the time of diagnosis. Fentiman *et al* (2005) reported that in a series of 166 women with stage 1 or 2 invasive breast cancer, smoking was the third most important predictor of distant relapse-free breast cancer-specific and overall survival after stage and age at diagnosis. A large overview of 53 epidemiological studies (Collaborative Group on Hormonal Factors in Breast Cancer, 2002) found that smoking had little or no effect on the risk of initially developing breast cancer.

The data in our study demonstrated a marginally significant association between mastectomy as the final breast status and the occurrence of systemic metastases. Most of the reasons for such an association should be linked to tumour variables such as size, grade and nodal involvement. The association after we had taken into account the influence of those tumour variables is not obviously explained, although it is possible that it is linked with socio-economic deprivation. The area serving the Leeds Teaching Hospitals Trust has a population with relatively high levels of deprivation, and previous work in both Scotland (Thomson *et al*, 2001, 2004) and Yorkshire (Downing *et al*, 2007) has shown that deprived women are, especially those aged under 65 years at diagnosis, more likely to undergo a mastectomy than WLE. These studies also showed that deprivation was associated with poorer survival, so it is reasonable to assume an association with the occurrence of systemic recurrences, although we have not specifically examined this in our study. It is also possible that the association between final breast surgery and the occurrence of systemic recurrences is associated with other tumour-associated characters not adjusted for, or is in some other way related to the greater magnitude of surgery involved in a mastectomy than WLE. Other studies have shown increased rates of wound complication rates with axillary surgery, however our study did not demonstrate this.

While these data do represent a hospital series for a single surgeon within an NHS Teaching Hospitals Trust, the mastectomy and breast conservation rates are similar to other regional data presented at the annual audit by The Association of Breast Surgery at BASO 2006 (*Breast Cancer Clinical Outcome Measures Newsletter*, 2006, **1**(1)). It is difficult to obtain comparative published data of total series of patients, which are just not related to patients involved in clinical trials. The data for the study were obtained by retrospective case note review. Patients were followed closely subsequent to their surgery, and all were seen at clinics within a short time of hospital discharge to discuss their tumour histology and plan adjuvant therapy. It is unlikely, therefore, that there is significant inaccuracy in the recording of whether patients had wound complications. Indeed, data retrieval was independently checked using a random sample of cases, which were re-examined, and it was found to be consistent.

The literature on the influence of wound complications on cancers at other anatomical sites is sparse and inconsistent. Improved survival associated with postoperative wound infection was reported in laryngeal cancer by Schantz *et al* (1980) and Ramadan and Witmore (1992).

In contrast Jackson and Rice (1990), Grandis *et al* (1992) and Rodrigo and Suarez (1998) reported adverse effects on survival. Fucini *et al* (1985), Sauven *et al* (1989) and Kressner *et al* (2002) reported increased local recurrence in colorectal cancers after perineal wound infection similar to the report of Varty *et al* (1994). They found no difference in overall survival and concluded that there was no overall survival difference for patients with or without postoperative sepsis following excision of colorectal cancer. This is in contrast to Fujita *et al* (1993), who report an increased incidence of local recurrence as well as poor prognosis. In malignant melanoma, wound infection at the site of nodal dissection was associated with fewer nodal recurrences but no change in survival (Papachristou and Fortner, 1979). Chang *et al* (2005) recently reported further studies on wound healing characteristics of fibroblasts and the link to cancer progression. They found an association between a ‘wound response signature’ and increased metastases in a series of early breast cancer patients.

Many cytokines and chemokines are released during wound healing and inflammation: TNF*α*, interleukin 6, PDGF and VEGF to cite a few. Activated macrophages, fibroblasts, smooth muscle cells and keratinocytes release them along with other inflammatory cytokines. In the experimental setting, TNF*α* acts as a tumourigenic agent and tumour promoter (Coffey *et al*, 2003). Various other mechanisms explain how other factors like VEGF, PDGF and interleukins influence tumour growth (Dvorak, 1986; Dvorak *et al*, 1995; Abramovich *et al*, 1999; Balkwill and Mantovani, 2001; Coussens and Werb, 2002; Chang *et al*, 2005). There is also evidence to suggest that cell-mediated immunity and natural killer cell function is suppressed by the effect of anaesthesia and the post-surgical stress response (Bogden *et al*, 1997). If this ‘window period’ of decreased immunity is prolonged by wound complications like flap necrosis and breakdown, there may be increased growth of occult micro metastases. It is not possible to state from this study if those influences are due to altered immun response or active promotion of circulating or micrometastatic tumour cells or some other mechanisms. Retsky *et al* (2005) suggest ‘surgery-induced angiogenesis’ as an important cause of the frequently noted early peak in recurrence rates after breast cancer excision. They suggest that therapeutic strategies might allow for this effect by the introduction of anti-angiogenetic drugs at the time of surgery.

It is of interest that the increase in systemic recurrence rate subsequent to wound complication was seen across all three prognostic NPI groups and was of a similar magnitude (Table 3). This would suggest that intrinsic tumour characteristics reflecting tumour aggression are not those most influenced by wound factors.

## CONCLUSIONS

The results of this study suggest that delayed wound healing is associated with an increased rate of systemic recurrence after primary breast cancer excisional surgery. As this study is only hypothesis generating and falls short of proving any cause and effect relationship, the statistically significant effect of having a wound complication in the multivariate analysis after adjustment for case mix does suggest that further studies, including prospective evaluation at more than one centre, are required to confirm the findings. If the relationship showing the considerable increase in systemic recurrence rates in patients who have wound complications at the time of their primary breast cancer surgery can be supported, then the implications on the surgical process are substantial. This is because the benefits to systemic recurrence rates and, ultimately, death, which could be achieved by reducing wound complications, are of similar magnitude to the known benefits seen for giving appropriate adjuvant treatment to women with early breast cancer. Therefore, there is an increased onus on the surgeon to minimise wound complication by attention to technique and choice of less risk-associated interventions in a multi-disciplinary environment, which may include preoperative anti-angiogenic therapies.

## Figures and Tables

**Table 1:** Number and percentage of patients with wound complications according to different factors

	**All patients**	**Wound complication**		
	**Number**	**Number**	**%**	***P*-value^a^**
*Age group at surgery (years)*				0.25
<50	294	21	7.1	
50–64	423	46	10.9	
65–74	212	16	7.5	
75+	136	10	7.4	
				
*Any co-morbidity*				0.18
Yes	287	31	10.8	
No	778	62	8.0	
				
*Current smoker*				0.001
Yes	230	34	14.8	
No	835	59	7.1	
				
*Diabetes*				0.03
Yes	35	7	20.0	
No	1030	86	8.3	
				
*ER/PR status*				0.22
Positive	857	70	8.2	
Negative	208	23	11.1	
				
*Node status*				0.37
Positive	487	49	10.1	
Negative	550	42	7.6	
Not known	28	2	7.1	
				
*NPI status* b				0.12
Good	361	27	7.5	
Intermediate	447	35	7.8	
Poor	228	29	12.7	
Not known	29	2	6.9	
Total	1065	93	8.7	
				
*Final breast surgery*				<0.001
Mastectomy	593	70	11.8	
Wide-local excision	472	23	4.9	
				
*Use of radiotherapy*				0.34
Given	748	61	8.2	
Not given	317	32	10.1	
				
*Use of chemotherapy*				0.64
Given	338	27	8.0	
Not given	727	66	9.1	
				
*Use of hormone therapy*				0.78
Given	879	76	8.6	
Not given	186	17	9.1	
Total	1065	93	8.7	

a *χ*^2^-test for association.

b When the 29 patients with NPI status unknown were excluded from the analysis, the association between NPI status and wound complication was marginally nonsignificant (*P*=0.06).

**Table 2:** Unadjusted^a^ and adjusted^b^ hazard ratios of a systemic recurrence with a median of 54 months follow-up, based on all cases (*n*=1065)

**Factor**	**No. of cases**	**Unadjusted hazard of systemic recurrence (95% CI)**	***P*-value^c^**	**Adjusted hazard of systemic recurrence (95% CI)**	***P*-value^d^**
*Wound complication occurred*			<0.0001		<0.0001
Yes	93	2.87 (1.97, 4.18)		2.52 (1.69, 3.77)	
No	972	1.0		1.0	
					
*Age group (years)*			0.35		0.78
<50	294	1.0		1.0	
50–64	423	0.75 (0.52, 1.07)		0.94 (0.63, 1.41)	
65–74	212	0.73 (0.47, 1.13)		1.18 (0.67, 2.08)	
75+	136	0.78 (0.47, 1.31)		1.21 (0.59, 2.49)	
					
*Any co-morbidity*			0.62		0.58
Yes	287	0.92 (0.65, 1.30)		0.89 (0.60, 1.34)	
No	778	1.0		1.0	
					
*Smoker*			0.14		0.03
Yes	230	1.29 (0.92, 1.81)		1.50 (1.04, 2.15)	
No	835	1.0		1.0	
					
*ER/PR status*			<0.0001		<0.0001
Positive	857	1.0		1.0	
Negative	208	2.97 (2.18, 4.04)		2.22 (1.45, 3.40)	
					
*NPI status* e			<0.0001		<0.0001
Good	361	1.0		1.0	
Intermediate	447	3.32 (1.89, 5.87)		2.59 (1.42, 4.72)	
Poor	228	13.35 (7.73, 23.04)		8.30 (4.28, 16.09)	
					
*Final breast surgery*			<0.0001		0.03
Mastectomy	593	1.0		1.0	
Wide-local excision	472	0.41 (0.29, 0.58)		0.63 (0.42, 0.96)	
					
*Use of radiotherapy*			0.002		0.07
Given	748	1.77 (1.23, 2.54)		1.50 (0.96, 2.34)	
Not given	317	1.0		1.0	
					
*Use of chemotherapy*			<0.0001		0.73
Given	338	2.79 (2.07, 3.77)		1.09 (0.68, 1.73)	
Not given	727	1.0		1.0	
					
*Use of hormone therapy*			0.008		0.96
Given	879	0.62 (0.43, 0.88)		1.01 (0.63, 1.63)	
Not given	186	1.0		1.0	

a Derived from univariate Cox's proportional hazards models.

b Derived from a multivariate Cox's proportional hazards model.

c *P*-values for the factors are the Wald statistics for the estimates in each univariate model.

d *P*-values for the factors are the Wald statistics for the estimates in the model, conditional on the other factors being present.

e Twenty-nine patients with NPI status not known were excluded.

**Table 3:** Numbers having systemic recurrence, with adjusted^a^ hazard ratios of a systemic recurrence with a median of 54 months follow-up, based on all cases with a known NPI status (*n*=1036)

	**Patients not having a wound complication**			**Patients having a wound complication**		
	**No systemic recurrence/total number**	**%**	**Adjusted hazard of systemic recurrence (95% CI)**	**No systemic recurrence/total number**	**%**	**Adjusted hazard of systemic recurrence (95% CI)**
*NPI status*						
Good	11/334	3	1.0	4/27	15	3.5 (1.1, 11.3)
Intermediate	48/412	12	2.8 (1.4, 5.5)	10/35	29	7.1 (2.9, 17.1)
Poor	75/199	38	9.1 (4.4, 18.8)	18/29	62	21.3 (8.9, 50.8)

a Derived from a multivariate Cox's proportional hazards model.
